# Hierarchically structured activated carbon for ultracapacitors

**DOI:** 10.1038/srep21182

**Published:** 2016-02-16

**Authors:** Mok-Hwa Kim, Kwang-Bum Kim, Sun-Min Park, Kwang Chul Roh

**Affiliations:** 1 Energy and Environmental Division, Korea Institute of Ceramic Engineering and Technology, Jinju 660-031, Republic of Korea; 2 Department of Materials Science and Engineering, Yonsei University, Seoul 120-749, Republic of Korea

## Abstract

To resolve the pore-associated bottleneck problem observed in the electrode materials used for ultracapacitors, which inhibits the transport of the electrolyte ions, we designed hierarchically structured activated carbon (HAC) by synthesizing a mesoporous silica template/carbon composite and chemically activating it to simultaneously remove the silica template and increase the pore volume. The resulting HAC had a well-designed, unique porous structure, which allowed for large interfaces for efficient electric double-layer formation. Given the unique characteristics of the HAC, we believe that the developed synthesis strategy provides important insights into the design and fabrication of hierarchical carbon nanostructures. The HAC, which had a specific surface area of 1,957 m^2^ g^−1^, exhibited an extremely high specific capacitance of 157 F g^−1^ (95 F cc^−1^), as well as a high rate capability. This indicated that it had superior energy storage capability and was thus suitable for use in advanced ultracapacitors.

Ultracapacitors (UCs) show great promise as energy storage devices because of their high power and long cycling life. Typically, UCs are based on the accommodation of electrochemical charge in the electric double layer^1^,^2^. Therefore, to ensure a high specific capacitance, the specific surface area of the electrode material used should be as high as possible, such that a large number of electrolyte ions can be accommodated at the electrode/electrolyte interface, resulting in a high electric double-layer capacitance. Consequently, the performance of UCs depends significantly on the electrode material used; they should ideally have a high specific surface area and a large pore volume, as these aid electrolyte-ion transport in the pore channels^1^,^2^,^3^,^4^. Most commercial UCs employ activated carbon (AC) electrodes, owing to their high specific surface area, excellent electrochemical properties, and relatively low costs^5^. However, AC has a wide pore distribution and contains micropores and macropores, as well as random pore connections. The intrinsically irregular pores of AC limit its accessibility to electrolyte ions during the charge/discharge process in the case of organic ions for non-aqueous systems, owing to the fact that they are closed or that the passages between them are narrow^5^,^6^,^7^,^8^. Thus, adequate control over the specific surface area and pore size of electrode materials is crucial, because a high microporosity dramatically decreases the capacitance at large current densities and limits the power density of UCs, especially in non-aqueous electrolytes.

Mesoporous carbon materials are also used as electrode materials for UCs, owing to their narrow pore size distribution, which allows for rapid ion diffusion and mass transfer^9^,^10^,^11^. Moreover, the use of mesostructured silicates as a hard template material allows for the synthesis of well-ordered porous carbon materials with tailored structural characteristics, including controlled pore size, pore volume, particle size, and morphology^12^. Such mesoporous carbon materials usually exhibit better electrochemical performance than those of AC at high current densities, because of the presence of mesopore channels and interconnections, which provide suitable paths for the penetration and transportation of electrolyte ions. However, the degree of improvement in the specific capacitance of mesoporous carbon materials has been low, owing to their relatively low surface area.

Several studies reported an important capacitive contribution from micropores^1^,^2^,^3^,^4^,^5^. The macroporous, mesoporous, and microporous structures have an enhanced electrochemistry performance compared with that of the single-sized pore materials. As a result, the macropores minimize the ion diffusion distances to the interior surfaces, the mesoporous provide low resistant pathways for the ions through the porous particles, and the micropores strengthen the electric double layer capacitance. Given the high specific capacitance of microporous materials and the high rate capability of mesoporous ones, a potential approach for improving the power performance of UCs is to increase the number of ion-transport paths present within the microporous carbon by producing mesoporous channels inside the carbon particles. Thus, hierarchically porous carbon materials have come to be regarded as being highly suited for UCs, owing to their low resistance to electrolyte diffusion and their high energy density^3^,^4^,^5^,^6^,^7^. Wang *et al.*^13^ synthesized a carbon material suitable for high-power-density UCs. The excellent properties of this material were attributable to the presence of three-dimensional hierarchical carbon, which was both porous and graphitic in nature. They determined that both the surface area and the porosity of a material determine its suitability for use as an electrode material in UCs and that the presence of both macropores and mesopores is a prerequisite for the fast transport of electrolytes to the smaller mesopores and micropores. However, the process for producing such materials is complicated and involves several multistep procedures. Though significant progress has been made in the design of porous carbon materials, further advances are needed for the rational design of materials with the desired porous characteristics.

Herein, we report a novel approach for producing hierarchically structured activated carbon (HAC). This approach involves the synthesis of a mesoporous silica template/carbon composite and its subsequent chemical activation. Typically, the silica matrix is dissolved through a treatment with hydrofluoric acid. In contrast, we employed the chemical activation method to remove the silica matrix; this increased the volume of the micropores. The resulting HAC nanostructure, that is, the designed hierarchical structure, which consisted of macropores as well as mesopores and micropores, not only contained accessible pathways for ion transport but also exhibited a surface area high enough for ion adsorption/desorption. The unique structure of the HAC makes it a promising material for UCs, as it exhibited both a high capacitance and excellent rate capability. Hence, this study provides new insights into the design of ideal materials for UCs that exhibit both high energy capacities and high power densities.

## Results

**Figure 1:**
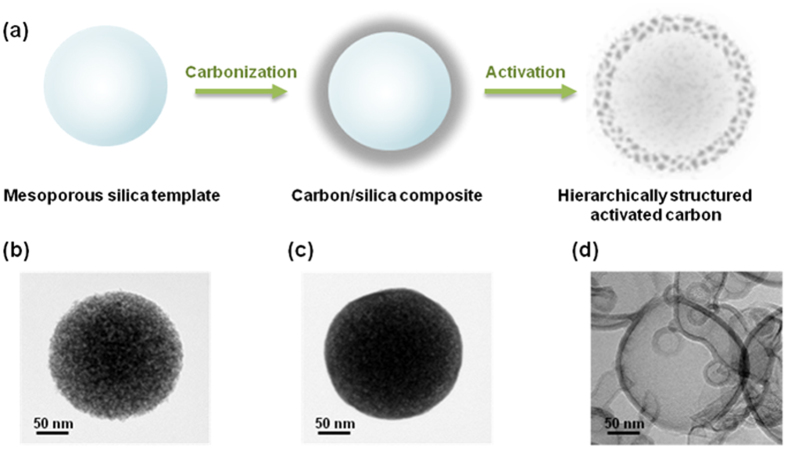
(**a**) Illustration of the method used for synthesizing the HAC. HRTEM images of the (**b**) mesoporous silica template, (**c**) carbon/silica composite, and (**d**) HAC.

The synthesis method used, shown schematically in Fig. 1a, allowed HAC to be produced in two simple steps using pitch as the carbon source. In brief, spherical mesoporous silica was used as a hard template material. The pitch such as carbon precursors is infiltrated into template pores in a liquid form by dissolving in tetrahydrofuran (THF). A driving force for the migration of carbon-containing molecules into pores has a capillary effect that occurs only when a proper attractive interaction exists between hydroxyl- groups present on the surface of silica pore walls and the aforementioned molecules. The mesoporous silica spheres and pitch were carbonized to prepare the carbon/silica template. After carbonization, the sample was activated using KOH, resulting in HAC with a hierarchical porous structure and spherical morphology. Figure 1b–d show HRTEM images of the silica template, the carbon/silica composite, and the HAC spheres, respectively. The core/shell and porous structures are well defined.

**Figure 2:**
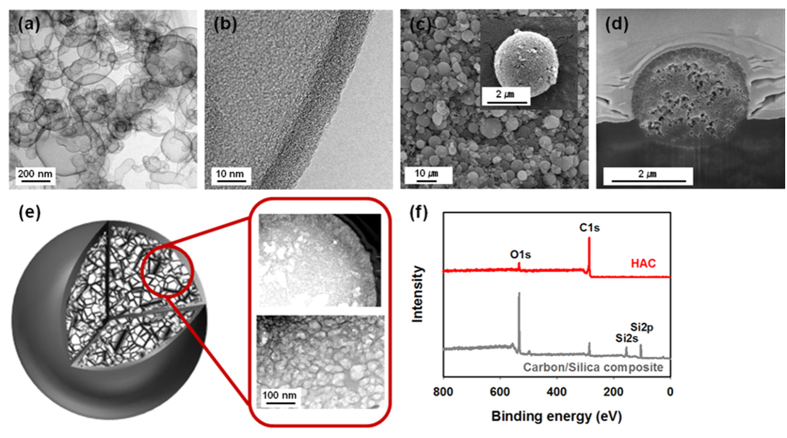
(**a**,**b**) HRTEM images of the HAC sample at different magnifications, showing its core/shell structure and spherical morphology. The thickness of the HAC shells was approximately 10 nm. (**c**) FESEM images of the HAC sample. The inset in (**c**) shows a high-magnification image of a HAC sphere. (**d**) SEM image of a focused-ion-beam-cut cross-section of a HAC sphere. (**e**) Schematic representation of the hierarchical porous structure of the HAC spheres, which had a foam-like core and a dense shell. Also shown are HRTEM images of the HAC cross-section. (**f**) Wide-scan XPS spectra of the carbon/silica composite and the HAC sample.

The typical HRTEM images of the HAC sample indicated that it had a spherical morphology and consisted of particles of non-uniform size. It was also observed that the HAC possessed a core/shell structure, as shown in Fig. 2a,b. The particle size was non-uniform, because it was dependent on the size of the particles of the mesoporous silica template (Fig. S1). After the activation process, the spherical morphology was largely maintained; however, a few small particles aggregated or became damaged (Fig. 2a). The HAC cores had the same diameter as that of the silica template particles, while the shell thickness was approximately 10 nm (Fig. 2b). The shells consisted of a number of graphene-type layers oriented in different directions; this indicated that the HAC sample had a disordered shell structure. Micropores were observed in the carbon shells, possibly owing to the collapse of the carbon when the mesoporous silica was removed during the chemical activation process. This happened probably because of the penetration of potassium into the carbon/silica composite structure during the chemical activation process and the subsequent removal of the silica template, which resulted in the formation of HAC structures consisting of meso-/macroporous cores and microporous shells. The FESEM images of the particles show clearly that they had a spherical morphology and that their surfaces contained pores (Fig. 2c). To elucidate the reason for the presence of pores on the surfaces of the particles, their internal structures were observed by performing SEM imaging on a sample cross-section cut with a focused-ion beam. Numerous pores were evident in the magnified images of the cross-section (Fig. 2d), confirming the nanoporous internal structure of the HAC sample. A schematic illustration of the hierarchically ordered porous structure is shown in Fig. 2e. The meso-/macroporous network, including the microporous shells, can be seen in the image. Further information regarding the internal structure of the HAC was obtained by performing HRTEM on the sample cross-section. The obtained images also confirmed that the HAC had a meso-/macroporous structure and microporous shells. Further, the TEM images showed clearly that the foam-like HAC had a porous, net-like internal structure. The XPS spectrum of the HAC sample confirmed that the silica template was completely removed after the chemical activation process (Fig. 2f). This was further confirmed by thermogravimetric analysis (TGA) in Fig. S2. This indicates that silica was removed during chemical activation.

When the silica is removed by KOH, KOH will chemically react with silica by the following equation:^14^


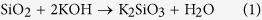


**Figure 3:**
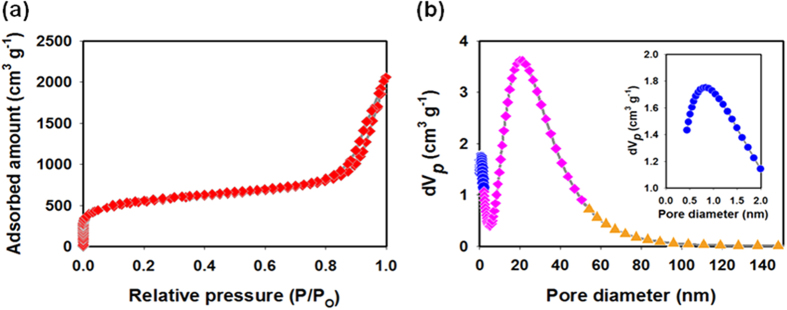
(**a**) Nitrogen adsorption–desorption isotherms of the HAC. (**b**) Pore-size distribution curves of the HAC, as determined using the NLDFT model. The inset shows the size-distribution curves of the HAC micropores.

As shown in Fig. 3, the N_2_-sorption isotherms confirmed the high porosity of the HAC sample, as it exhibited a high nitrogen uptake capacity. The specific surface area as measured by the BET method was 1,957 m^2^ g^−1^, while the total pore volume was 3.0 cm^3^ g^−1^. The pore-size distribution, derived using the nonlocal density functional theory (NLDFT), is shown in Fig. 3b. Most of the pores were mesopores (approximately 20 nm in size); however, micropores were also present in the hierarchically ordered, nanostructured carbon^15^,^16^. The HAC sample exhibited a hierarchical nanostructure with a macroscopic core, with mesopores and micropores being present in the microporous shell. These unique structural features suggest that it should be suitable for use in UCs.

The hierarchical structure within the carbon nanonetwork should allow for the rapid transport and diffusion of electrolyte ions throughout the entire surface area, while the high surface area, which is primarily attributable to the micropores in the carbon nanoparticles, should aid charge accumulation. Next, we determined whether the HAC sample exhibited potential as an electrode material for advanced UCs. To evaluate the potential of the HAC sample for use as an electrode material, it was used to assemble a two-electrode system. Further, to demonstrate the structural advantages of the HAC, a commercially available AC sample with a similar specific surface area (2,049 m^2^ g^−1^) was also tested for comparison (Fig. S3).

**Figure 4:**
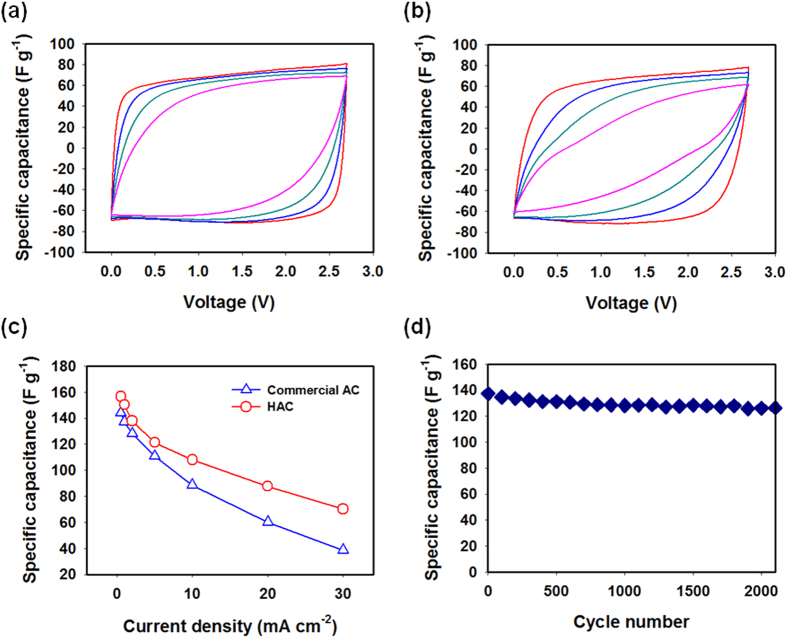
CV profiles of (**a**) the HAC and (**b**) a commercial AC for 2–20 mV s^−1^. (**c**) Plot of the gram-wise specific capacitance of the HAC and the commercial AC at different current densities. (**d**) Cycling performance of the HAC at a current density of 1.0 mA cm^−2^.

As shown in Fig. 4a, the CV profile of the HAC sample was nearly rectangular at all sweep rates between 2 and 20 mV s^−1^. In contrast, that of the commercial AC was distorted (Fig. 4b). These results indicated that the HAC sample exhibited good capacitive behaviour, allowing for ready charge propagation and easy ion transport^17^,^18^,^19^. In particular, with an increase in the sweep rates (e.g., at 20 mV s^−1^), the differences in the shapes of the CV curves become more significant, demonstrating that the HAC exhibited better ion transportability at high charge/discharge rates. Moreover, it was confirmed the current output of the HAC was larger than that of the commercial AC; this was indicative of the larger capacitance of the HAC. The electrical conductivity of HAC and commercial AC was measured by a typical two-probe method. The results show that HAC displayed much higher conductivity (5.4 S cm^−1^) than did commercial AC (2.6 S cm^−1^), which was in good agreement with the CV curve (Fig. S4).

The specific capacitance of the HAC was calculated from its galvanostatic charge/discharge curves. As shown in Fig. 4c, the rate performance was assessed from the plots of the specific capacitance with respect to the current density. The gram-wise specific capacitances obtained using the HAC were 157, 150, and 138 F g^−1^ at current densities of 0.5, 1.0, and 2.0 mA cm^−2^, respectively. Note that despite this decrease in the specific capacitance with an increase in the current density, 45% of the capacitance at 0.5 mA cm^−2^ was retained at 20.0 mA cm^−2^. The specific capacitance of the HAC is compared with that in the literature^20^. As a result, a superior pore structure feature of the HAC is that the high rate capability, as compared to the literature.

Because a loss in specific capacitance is usually related to the reaction kinetics at the electrode, this increase indicated that an electrical double layer was generated in the surface of the HAC more rapidly. Considering the similarity in the surface areas of the HAC sample and the commercial AC, it can be concluded that the nanopores within the unique nanonetwork-like structure of the HAC could be accessed by electrolytes more readily and thus had a significantly higher utilization rate than did those in the commercial AC. The HAC sample also exhibited highly stable cycling performance (Fig. 4d), undergoing only a small loss in capacitance after 2,000 cycles at a current density of 1.0 mA cm^−2^. Its Coulombic efficiency remained at more than 99%. The superior rate capability and excellent capacitive behaviour of the HAC can be attributed to the effectiveness of the reaction occurring on its surface and to its high electrical conductivity.

## Discussion

In this study, we prepared HAC by synthesizing a mesoporous silica template/ carbon composite and subsequently chemically activating it. The synthesized HAC had a well-designed, unique porous structure, which allowed for large interfaces for the efficient formation of an electric double layer. The spherical particle morphology as well as the hierarchical macroporous and mesoporous structural network survived the chemical activation process, guaranteeing the rapid transfer and diffusion of the electrolyte ions. Given the unique characteristics of the HAC, we believe that the developed synthesis strategy provides important insights into the design and fabrication of hierarchical carbon nanostructures. Moreover, this work should result in the development of high-performance energy storage devices.

## Methods

### Material preparation

The HAC sample was prepared from the pitch (Anshan pitch, Anshan Chemical Co. Ltd., China); mesoporous silica (ABC nanotech Co. Ltd., Korea) was used as a hard template material. First, 15.0 g of pitch was dissolved in tetrahydrofuran (THF) (Sigma Aldrich). Next, 1.0 M sulfuric acid (H_2_SO_4_) was added to the solution and stirred for 12 h. Then, 10.0 g of mesoporous silica was added to the solution under stirring at room temperature. Next, the THF was removed through evaporation at room temperature under mild stirring. After all the THF had been removed through evaporation for 5–8 h, the remaining product was dried at 120 °C for 24 h. The resulting powder was carbonized at 900 °C for 1 h in a N_2_ atmosphere, yielding the silica/carbon composite. Finally, the silica/carbon composite was mixed with small flakes of potassium hydroxide (KOH). The KOH-based activation process involved heating the mixture at 900 °C for 1 h in an Ar flow; the composite/KOH ratio was 1:4 by weight. The alkali-rich residual carbon was neutralized with a hydrochloric acid (HCl) solution, filtered, and washed with distilled water until the pH was 6–7.

### Characterization

The pore structure of the HAC sample was determined from its N_2_ adsorption–desorption isotherms, which were obtained using a gas analyser (Belsorp-Mini II, Japan). The sample was outgassed at 300 °C for 18 h under vacuum prior to the gas adsorption measurements. The specific surface area was calculated using the Brunauer–Emmett–Teller (BET) method. The morphologies and structures of the various samples were characterized using field-emission scanning electron microscopy (FESEM) (JEOL, JSM-7000 F, Japan) and high-resolution transmission electron microscopy (HRTEM) (JEM 2000EX, JEOL, Japan). The atomic concentrations were determined by X-ray photoelectron spectroscopy (XPS), which was performed at 1.1 × 10^−7^ Pa using a PHI 5000 VersaProbe system and Al-Kα radiation (ULVAC-PHI, Inc., Japan). Thermogravimetric analysis (TGA) of the HAC was performed using NETZSCH TG 209 F3 (heating rate of 10 °C min^−1^) in air conditions. Electrical conductivity of the samples in a Teflon cylinder cell filled with the powder was measured using a current–voltage (I-V) source. Here, 20 mm-diameter electrodes were used as probes, and the conductivities of the samples were calculated from the slope of I-V curves because I-V characteristics were ohmic in the measured voltage range (from 0 to 5 mV).

### Electrochemical measurements

Rubber electrodes were fabricated from the HAC sample using polytetrafluoroethylene (D-60, Daikin Industries, Japan) as the binder and Super-P black (MMM Carbon Co., Belgium) as the conducting agent. The electrodes were composed of 90 wt% active material, 5 wt% conducting material, and 5 wt% binder. The prepared electrodes were punched with a 12-mm-diameter punch and dried in a vacuum oven. For comparative purposes, electrodes were also prepared in the same manner using as the active material a commercial AC (CEP21, Power Carbon Technology, Korea). The density of the resulting electrode exhibits 0.68 g cm^−3^ (HAC) and 0.57 g cm^−3^ (commercial AC).

The electrochemical characteristics of the electrodes were examined using type-2032 stainless-steel two-electrode cells containing 1.0 M tetraethylammonium tetrafluoroborate in propylene carbonate as the electrolyte. The stainless-steel coin cells, which had two symmetrical electrodes separated by a porous polymer, were assembled in an Ar-filled glovebox. Galvanostatic charge/discharge tests were performed using a battery tester (Maccor, Series 4000, USA). Cyclic voltammetry tests were performed for voltages of 0–2.7 V using a potentiostat (EC-Lab, France). All the electrochemical tests were performed at room temperature. The specific capacitance of a single electrode in the symmetric two-electrode cell was calculated from the galvanostatic charge/discharge curves using the following equation:


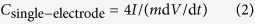


where *C*_single-electrode_, *I*, *m*, and d*V*/d*t* represent the specific capacitance of a single electrode, the constant applied current, the mass of the active material in the two electrodes, and the slope obtained from the discharge curve, respectively.

## Additional Information

**How to cite this article**: Kim, M.-H. *et al.* Hierarchically structured activated carbon for ultracapacitors. *Sci. Rep.* **6**, 21182; doi: 10.1038/srep21182 (2016).

## Supplementary Material

Supplementary Information
