# Supplementary material for: Hierarchically structured activated carbon for ultracapacitors
Source: Sci Rep. 2016 Feb 16;6:21182. doi: 10.1038/srep21182 (PMC4754731; doi:10.1038/srep21182)
Supplement: Supplementary Information [file srep21182-s1.doc]

**Hierarchically structured activated carbon for ultracapacitors** Mok-Hwa Kim*^a,b^*, Kwang-Bum Kim*^b^*, Sun-Min Park*^a^*, Kwang Chul Roh******^a^*

*^a^*Energy and Environmental Division, Korea Institute of Ceramic Engineering and Technology, Jinju 660-031, Republic of Korea

*^b^*Department of Materials Science and Engineering, Yonsei University, Seoul 120-749, Republic of Korea

Table S1. Pore characterization of HAC compared with a commercial AC (CEP21, Power Carbon Technology, Korea).

| Sample | S_BET_^[a]^ | V_T_^[b]^ |
| --- | --- | --- |
| HAC | 1957 | 3.0 |
| CEP21 | 2049 | 0.9 |

[a] Specific surface area (m^2^·g^-1^) [b] Total pore volume (cm^3^·g^-1^)

Table S2. Rate capability of HAC compared with a commercial AC and literature.

| Sample | S_BET_^[a]^ | MAX.SC^[b]^ | Min.SC^[c]^ |
| --- | --- | --- | --- |
| HAC | 1957 | 157 (at 0.5 mA·cm^-2^) | 70 (at 30 mA·cm^-2^) |
| CEP21 | 2049 | 144 (at 0.5 mA·cm^-2^) | 39 (at 30 mA·cm^-2^) |
| Ref.20 | 1778 | 150 (at 0.35 mA·cm^-2^) | ~40 (at 35mA·cm^-2^) |

[a] Specific surface area (m^2^·g^-1^) [b] Maximum specific capacitance (F·g^-1^) [c] Minimum specific capacitance (F·g^-1^)


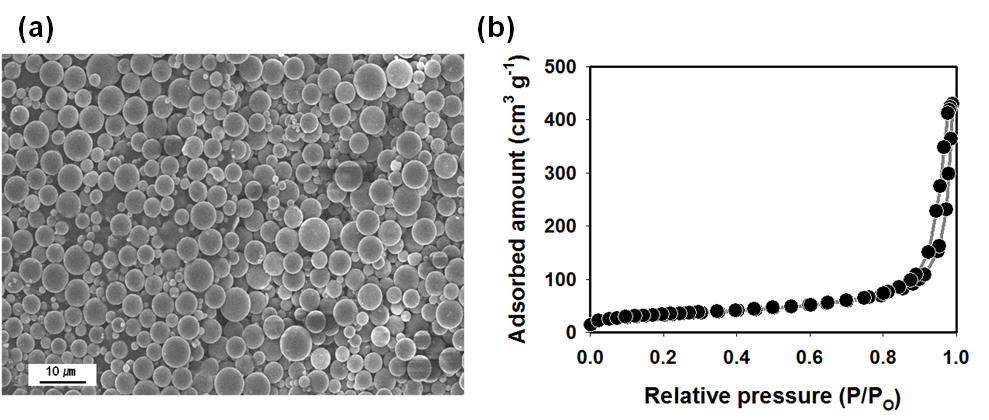


Fig. S1. (a) FESEM image and (b) Nitrogen adsorption–desorption isotherms of the mesoporous silica hard template.


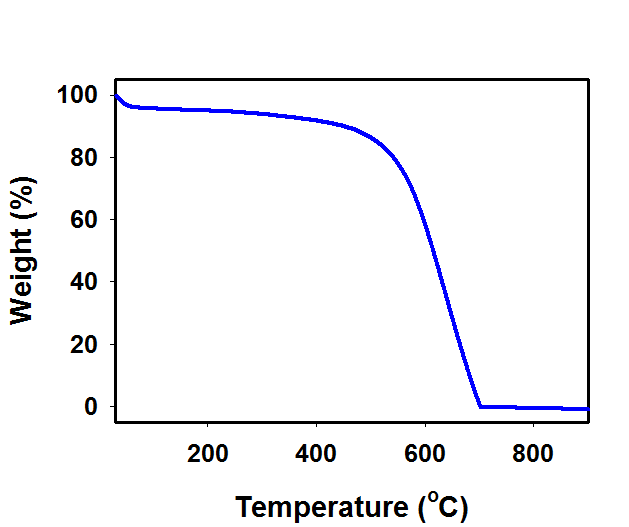


Fig. S2. TGA curve of HAC in air.


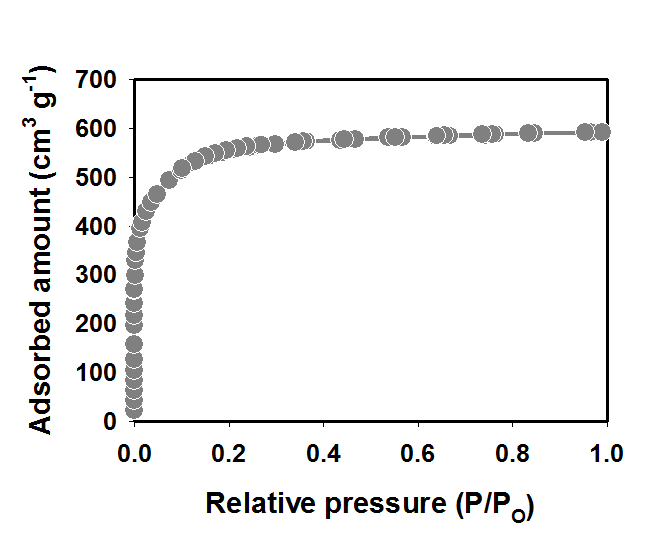


Fig. S3. Nitrogen adsorption–desorption isotherms of the commercial AC.


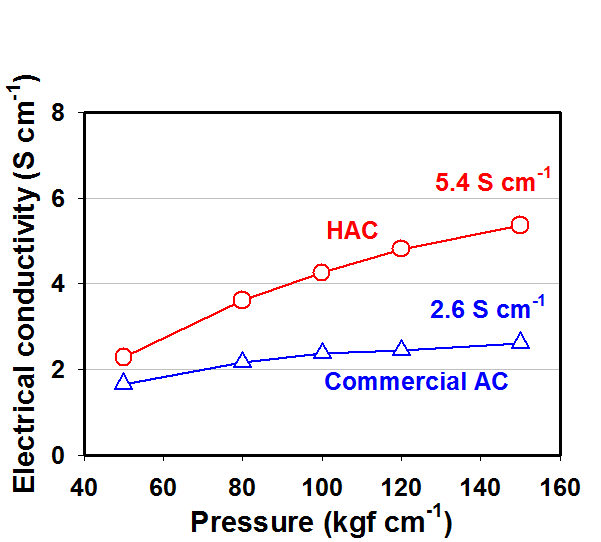


Fig. S4. Electrical conductivity of HAC (red) and commercial AC (blue) under various compressive pressure.

.
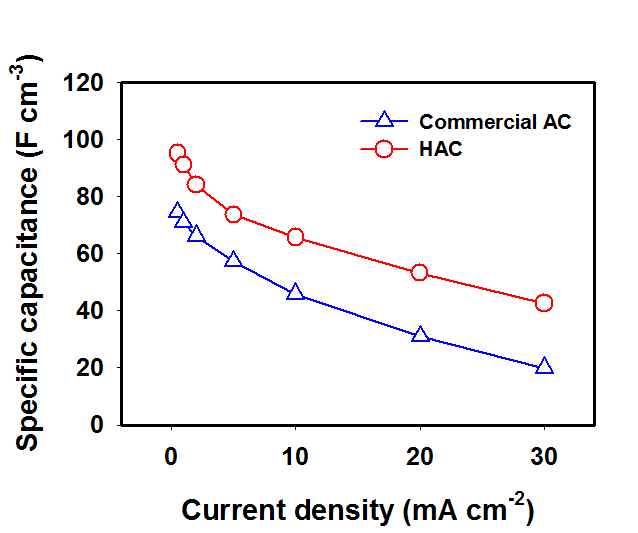


Fig. S5. Volumetric specific capacitances of the HAC and the commercial AC at different current densities.
